# Editorial: Integrative endothelial signaling mechanisms in resistance arteries

**DOI:** 10.3389/fphys.2026.1940175

**Published:** 2026-08-25

**Authors:** Daniel R. González, Xavier F. Figueroa

**Affiliations:** 1 Laboratory of Pharmacology and Physiology, Department of Basic Biomedical Sciences, Faculty of Health Sciences, Universidad de Talca, Talca, Chile; 2 Facultad de Ciencias Biológicas, Pontificia Universidad Católica de Chile, Santiago, Chile

**Keywords:** communication in the vessel wall, conducted vasodilation, control of vasomotor tone, endothelial cell heterogeneity, endothelial cells, nitric oxide signaling, S-nitrosylation in resistance arteries

The cardiovascular system is a complex network devised to provide blood-borne energy-and nutritional-related substrates to every single cell of the organism, in order to maintain homeostasis. For this task, fine control of blood flow distribution by resistance arteries is essential and endothelial cells play a pivotal role connecting tissue metabolic demands with the regulation of blood flow in the microcirculation (Bagher and Segal, 2011; Lillo et al., 2012; Segal, 2015). Therefore, signaling mechanisms of intercellular communication are fundamental to integrate these functions in the wall of resistance arteries and throughout the microvascular network (Segal and Jacobs, 2001; Figueroa and Duling, 2009).

In addition to endothelial cells, the wall of resistance vessels is primarily composed of smooth muscle cells and perivascular nerves (sympathetic and sensitive nerve terminals). These cells are organized in a multifaceted spatial architecture, where endothelial cells form a single-cell layer that lines the lumen of the vessels (i.e., the endothelium), while smooth muscle cells surround this inner endothelial layer (Davis et al., 1986; Westcott and Segal, 2013). Although the endothelium is physically separated from the smooth muscle by the internal elastic lamina (IEL), in resistance arteries, either endothelial or smooth muscle cells can project cellular extensions through the IEL to reach the other cell type at discrete points of contact known as myoendothelial junctions (MEJ), which are signaling microdomains (Sandow et al., 2003; Kerr et al., 2012). Interestingly, communication via gap junctions is not only restricted to the endothelial and smooth muscle cell layers, as these intercellular channels have also been found at MEJ (Sandow et al., 2003; Figueroa and Duling, 2009). As a result, in addition to paracrine signaling, gap junctions are key for coordinating functions across the wall of resistance arteries, through direct transfer of electrical signals and second messengers such as Ca^2+^ or inositol trisphosphate (IP_3_) between endothelial and smooth muscle cells, as well as among these two cell types (Figueroa et al., 2004; Figueroa and Duling, 2009). The intention of this Research Topic was to highlight the relevance of endothelial cell signaling in the integrative control and coordination of vasomotor tone in resistance arteries, which can be appreciated in this article collection that comprises four articles in total: one Review article and three original contributions.

Endothelial cells are essential for vascular homeostasis but also play a central role in controlling blood flow distribution through the regulation of vasomotor tone along different segments of resistance arteries, and in the maintenance of microvascular integrity by forming new vessels through coordinated cell migration and proliferation during angiogenesis (Lamalice et al., 2007; Figueroa and Duling, 2009; Espinoza and Figueroa, 2023). In addition, electrical signals that coordinate changes in vasomotor tone in the microcirculation are propagated through endothelial cells (De Wit et al., 2000; Figueroa and Duling, 2008; Márquez et al., 2023). Consistent with these functions, endothelial cells exhibit a strong metabolic and electric coupling mediated by gap junction channels (Figueroa et al., 2004; Figueroa and Duling, 2009). However, it has been found that endothelial cells show spatial and functional diversity, not only across different tissues but also within the same organ, revealing an unexpected functional differentiation of some specific endothelial cells (Han et al., 2025; Elsaid et al., 2026). This functional heterogeneity has been observed in the minute-to-minute control of vasomotor tone as well as in the long-term regulation of the endothelial monolayer (Bény et al., 2008; Han et al., 2025). It has been observed that the stimulation of intact arteries with endothelium-dependent vasodilators triggers an increase in intracellular Ca^2+^ concentration, but only in specific endothelial cells, which initiate a Ca^2+^ signal that rapidly spreads to neighboring cells via gap junctions (Ohi et al., 2001; Boittin et al., 2013). On the other hand, distinct proliferative programs across organ-specific endothelial cell subsets were recently described in a systematic analysis using a combination of RNA sequencing, spatial transcriptomics, and single-cell RNA sequencing (Han et al., 2025). Although the variability of endothelial cell membrane potential in resistance vessels has been previously reported (Siegl et al., 2005), the research article by Burboa et al. (Burboa et al.) represents the first systematic characterization of the influence of connexin channel-mediated communication on the electrical properties of different endothelial cell subpopulations. This study shows that the transcriptional and electrical identity of endothelial cells relies on the level of intercellular coupling. In this context, coupled endothelial cells were found to exhibit a heterogeneous but clearly hyperpolarized resting membrane potential, similar to that observed in intact resistance arteries (Siegl et al., 2005). In contrast, the membrane potential of isolated endothelial cells was more depolarized, and the hyperpolarization activated by the endothelium-dependent vasodilator acetylcholine (ACh) was blunted. Consistent with these findings, intercellular coupling was related to the upregulation of Ca^2+^-activated K^+^ channels of small (SK_Ca_) and intermediate conductance (IK_Ca_), as well as the inwardly rectifying potassium channel Kir2.1, which was associated with the expression of transcription factors like FOXO3 and MEF2C.

In addition to the transmission of electrical signals, endothelium-mediated signaling through nitric oxide (NO) is also important in regulating resistance vessel function within the microvascular network. NO production by endothelial cells leads to the relaxation of the underlying vascular smooth muscle cells through the activation of soluble guanylate cyclase and the cGMP/PKG pathway, which has been considered as the “classical” mechanism of NO signaling (Moncada et al., 1991; Moncada and Higgs, 2018). Nevertheless, in addition to activating soluble guanylate cyclase, S-nitrosylation (also termed S-nitrosation) has emerged as an important “non-classical” mechanism of NO signaling. It is crucial to note that, in contrast to the activation of the cGMP/PKG pathway, S-nitrosylation signaling is preferentially observed near the NO source, where NO concentration is higher (Qian et al., 2010; Martínez-Ruiz et al., 2012; Treuer and Gonzalez, 2015). S-nitrosylation involves NO-mediated oxidation of cysteine residues to form a nitrosothiol, a post-translational modification recognized for modulating the activity of several signaling proteins (Martínez-Ruiz et al., 2012; Pérez de la Lastra et al., 2026). As a physiological signaling mechanism, S-nitrosylation is transient, and the nitroso group can be removed through denitrosylation. Several non-enzymatic mechanisms of denitrosylation have been described, including reactions mediated by nucleophilic compounds, transition metal ions, and ascorbate (Majmudar and Martin, 2014; Stomberski et al., 2019; Pérez de la Lastra et al., 2026). However, this process has also been shown to be mediated enzymatically, which may contribute to limit and control the range of action of S-nitrosylation signaling to specific subcellular localization (Martínez-Ruiz et al., 2012; Pérez de la Lastra et al., 2026). In this context, two important enzymatic mechanisms of denitrosylation have been described: S-nitrosoglutathione (GSNO) reductase (GSNOR) and the thioredoxin system (Martínez-Ruiz et al., 2012; Stomberski et al., 2019). An interesting example of S-nitrosylation/denitrosylation signaling is the receptor-mediated activation of endothelial nitric oxide synthase (eNOS). This enzyme is tonically inhibited by S-nitrosylation (Ravi et al., 2004; Erwin et al., 2005), and the NO production observed in response to stimulation with vascular endothelial growth factor (VEGF) is facilitated by eNOS denitrosylation, followed by renitrosylation. Notably, subcellular localization is relevant for this signaling, as eNOS targeting to the cell membrane is required for its S-nitrosylation (Erwin et al., 2005, 2006). Therefore, NO-mediated S-nitrosylation has emerged as a relevant signaling mechanism involved in the minute-to-minute control of vascular function in conduit and resistance arteries, as described in detail by Lillo and Beuve (Lillo and Beuve). As noted in this article, the mechanisms of NO signaling are not uniform throughout the vascular tree, but vary significantly by vessel function. Primarily, conduit arteries, which function like low-resistance highways designed to transport blood efficiently to the tisues, and resistance arteries, which regulate blood flow distribution according to cellular metabolic demand. In conduit arteries, NO acts as an endothelium-dependent signal that diffuses to stimulate soluble guanylyl cyclase in smooth muscle cells, leading to cGMP production-mediated relaxation. In contrast, in resistance arteries, NO functions more as a redox signal, modulating key proteins such as connexin 43 and TRPV4 through S-nitrosation and transnitrosation.

Although NO is a potent vasodilator, the response activated by this endothelial signal is locally restricted. However, the regulation of blood flow distribution is a dynamic process that depends on the coordination of changes in diameter between different segments of the vascular resistance network (Figueroa et al., 2004; Segal, 2005; Figueroa and Duling, 2009; Bagher and Segal, 2011), which is likely achieved through the participation of a complementary vasodilator mechanism. In resistance arteries, the vasodilation induced by endothelial cell-mediated NO production is paralleled by an additional vasodilator mechanism associated with the endothelium-dependent hyperpolarization (EDH) of smooth muscle cells, through the activation of SK_Ca_ and IK_Ca_. Importantly, in blood vessels, both SK_Ca_ and IK_Ca_ are expressed only in endothelial cells, and the hyperpolarization mediated by the Ca^2+^-dependent activation of these K^+^ channels is transmitted to smooth muscle cells via gap junctions located at MEJ (Sandow et al., 2002; Eichler et al., 2003). While NO and EDH signaling contribute to the vasodilation activated at the site of stimulation, it is thought that only EDH participates in the integration of resistance artery function by initiating the longitudinal conduction of a hyperpolarizing signal. In this context, it is noteworthy that vasomotor signals generated at a particular arteriolar segment spread rapidly (<1 s) several millimeters along the vessel length, demonstrating functional coupling between distal and proximal sections of the vasculature (Beach et al., 1998; Gustafsson and Holstein-Rathlou, 1999; Bagher and Segal, 2011). Thus, the conduction of vasomotor signals along blood vessels contributes to the coordination of vascular function by integrating the changes in vasomotor tone of arterioles and feed arteries (Segal et al., 2000; Bagher and Segal, 2011). Gap junction communication between cells of the vessel wall provides a low-resistance intercellular pathway for the longitudinal transmission of this coordinated signaling (Figueroa and Duling, 2008, 2009; Jobs et al., 2012).

Changes in membrane potential of vascular smooth muscle cells play a central role in controlling vasomotor tone through modulation of voltage-dependent L-type Ca^2+^ channels (Jackson, 2000). Accordingly, depolarization is associated with vasoconstriction, while hyperpolarization is linked to vasodilation (Jackson, 2000, 2005). Direct measurements of membrane potential have shown that conducted vasomotor responses are associated with the propagation of an electrical signal, suggesting that the conduction of changes in diameter depends on the intercellular transfer of electrotonic potentials via gap junctions, from the stimulation site to distal domains (Xia and Duling, 1995; Pacicca et al., 1996; Welsh et al., 1998; Emerson and Segal, 2000). The electrotonic spread of current is a passive phenomenon and decays to 1/e (37%) of its initial magnitude over a length (length constant, λ) of a few millimeters. In the case of vasodilation triggered by endothelium-dependent agents such as ACh, the conducted vasodilation is thought to result from the electrotonic spread of the EDH-mediated hyperpolarization along the vessel length (Figueroa and Duling, 2009; de Wit et al., 2010). However, the characteristics of vasodilation initiated by endothelial cells are not consistent with this hypothesis, as endothelium-dependent vasodilator responses are propagated for several millimeters without apparent decay (Figueroa et al., 2007; Figueroa and Duling, 2008). Supporting the non-decremental spread of endothelium-mediated vasodilator responses, the work of Barrera et al. (Barrera et al.) in this article collection demonstrates that the vasodilation activated by ACh in a short arteriolar segment (local site, ~70 µm) is coupled to a mechanism that supports the regenerative propagation of the response along the entire length of the vessel. As expected, the ACh-induced vasodilation was partially mediated by NO production. Although the response initiated directly by NO was restricted to the stimulation site, a NO-dependent vasodilator component contributed to the response observed at the conducted sites, suggesting that the propagation of the vasodilator signal is associated with NO production along the vessel length. Interestingly, it was found that NO production has a dual role in the vasodilator response; in addition to contributing to the vasodilation, it also serves as a negative feedback signal of the regenerative mechanism of propagation.

The regulation and coordination of vasomotor tone in small arteries and arterioles of the microcirculation play a central role in controlling cardiovascular function, as most of the total resistance to blood flow resides on these vessels (Davis et al., 1986; Segal and Jacobs, 2001). Systemic blood pressure is determined by cardiac output and total vascular resistance to blood flow, and in turn, the control of blood flow distribution depends on the coordination of changes in diameter of feed arteries and arterioles (Segal et al., 2000; Segal and Jacobs, 2001). However, extrapolation of the mechanisms controlling the function of these vessels to human vascular physiology is not straightforward, as most studies analyzing the regulation of resistance arteries have been conducted using animals with identical genetical background as experimental models (e.g., mice C57bl/6J). This may introduce bias in assessing the relevance of the results concerning the progression of cardiovascular-related diseases. Therefore, parallel studies using outbred, genetically diverse experimental animals are necessary to validate the translational potential of new findings to human vascular physiology and pathophysiology, as described in the article by Looft-Wilson et al. (Looft-Wilson et al.). In this work, the authors performed a systematic analysis to assess the differences in vascular reactivity observed in mesenteric resistance arteries of outbred UM-HET3 mice compared to the standard inbred C57BL/6J mice. Although both strains exhibited similar characteristics in the vasoconstriction activated by phenylephrine and in the vasodilation evoked by ACh, the sensitivity to ACh was higher in UM-HT3 female mice. Additionally, this strain demonstrated a more pronounced myoendothelial feedback than that attained in C57 bl/6J mice, suggesting that UM-HT3 mice may be a more suitable model for certain translational vascular studies.

Overall, this Research Topic offers a comprehensive review and original contributions that further the investigation of endothelial signaling mechanisms controlling vasomotor tone in resistance arteries.
